# Social connectedness, optimism, and adaptive coping among university students in mainland China: a mediation model of perceived social support

**DOI:** 10.3389/fpsyg.2025.1693258

**Published:** 2025-11-17

**Authors:** Wen Fu

**Affiliations:** College of Marxism, Baoji University of Arts and Sciences, Baoji, Shaanxi, China

**Keywords:** social connectedness, optimism, perceived social support, adaptive coping, university students, mixed-methods, mediation, China

## Abstract

**Introduction:**

This mixed-methods study investigated the interplay among social connectedness, optimism, and perceived social support in predicting adaptive coping in university students in mainland China. The study examined the mediating role of perceived social support in the relationships of social connectedness and optimism with adaptive coping.

**Methods:**

Using an explanatory sequential design, quantitative data were first collected from 463 undergraduate students across three universities using questionnaires, and the hypothesized mediation model was tested using path analysis with structural equation modeling (SEM). Subsequently, qualitative data were gathered through semi-structured interviews with 20 students to explain the quantitative findings.

**Results:**

Quantitative results revealed that the hypothesized model showed a good fit to the data. Social connectedness and optimism were significant positive predictors of perceived social support and adaptive coping. Importantly, perceived social support significantly partially mediated the relationships of both social connectedness and optimism with adaptive coping. Qualitative findings contextualized these results, illustrating plausible mechanisms for the mediation pathways, while analysis of contradictory cases highlighted complexities such as optimism facilitating direct intrapersonal coping and pessimism potentially inhibiting support activation.

**Discussion:**

The study’s findings indicate that perceived social support plays a crucial, though not exclusive, mediating role associated with adaptive coping, through which social connectedness and optimism appear to exert some of their positive influences. These findings highlight the importance of fostering social support networks within universities to enhance student well-being and adaptive coping abilities.

## 1 Introduction

The university years are a critical developmental period marked by significant academic and social transitions that profoundly impact student well-being (Tanner et al., 2015). To thrive in this environment, students must navigate diverse stressors, including academic pressures and career uncertainty (Krypel and Henderson-King, 2010; Poole et al., 2023), making effective coping mechanisms paramount for psychological health (Lazarus and Folkman, 1984). Research has consistently shown that psychosocial resources are key predictors of positive outcomes in this population, but it is crucial to understand not just *which* resources matter, but *how* they work together to produce adaptive outcomes.

This investigation is situated within the unique psychosocial landscape of mainland China. Here, students navigate not only universal academic and social transitions but also intense cultural pressures related to high-stakes academic competition and strong family expectations (Yu and Luo, 2025; Zhao et al., 2015). Within this collectivist-influenced context, maintaining social harmony and interpersonal relationships is often prioritized (Markus and Kitayama, 1991). Consequently, a student’s sense of social connectedness and their perception of available support may be particularly powerful determinants of wellbeing. However, there may also be a cultural reluctance to explicitly burden others, making the *perception* of support—the subjective belief that help is available if needed—a more critical resource than the act of seeking it (Han and Pong, 2015; Ting and Hwang, 2009). Understanding how these culturally-salient social resources interact with internal, dispositional assets like optimism is therefore essential for supporting this student population.

This study conceptualizes these factors as a psychosocial system. We examine two foundational resources: social connectedness, a student’s external, relational asset (Baumeister and Leary, 1995), and optimism, an internal, dispositional asset (Carver et al., 2010). While prior studies have linked these factors independently to well-being (Chu et al., 2010; Malinauskas and Malinauskiene, 2020), their integrated influence on adaptive coping remains underexplored. This study addresses this gap by testing a specific mediation model. We hypothesize that the benefits of both social connectedness and optimism are channeled through a key psychological mechanism: perceived social support, the subjective appraisal of available aid from one’s network (Cohen and Wills, 1985; Zimet et al., 1988). We propose that these foundational assets converge on perceived social support, which in turn functions as the more proximal resource that facilitates adaptive coping. Understanding this pathway is critical for developing targeted interventions.

This research has both theoretical and practical significance. Theoretically, it offers a process-oriented model of how distal resources (connectedness and optimism) are translated into proximal coping behaviors. Practically, the findings can inform university interventions by highlighting that strengthening students’ perceptions of social support may be a powerful, centralized strategy for leveraging both relational and dispositional strengths to improve well-being and academic success.

The novelty of this study lies in its integrated mixed-methods approach. The quantitative phase uses structural equation modeling to rigorously test the hypothesized mediation model, while the qualitative phase uses semi-structured interviews to provide in-depth, contextual insights into the quantitative findings. This design offers a holistic interpretation that moves beyond statistical associations to capture the lived experiences underlying these psychosocial dynamics, particularly within the Chinese university setting. Ultimately, this study aims to provide empirical evidence for the mediating role of perceived social support, offering valuable theoretical insights and informing practical strategies to enhance the overall university experience for students.

## 2 Theoretical background and key constructs

### 2.1 Social connectedness: definition, outcomes, and challenges

Social connectedness is a multifaceted construct encompassing a subjective sense of belonging, interpersonal closeness, and integration within social networks (Baumeister and Leary, 1995; Lee and Robbins, 1998). As a fundamental human need (Baumeister and Leary, 1995), it reflects perceptions of acceptance and meaningful involvement. It is essential to distinguish this construct from perceived social support, which is the hypothesized mediator in our model. While social connectedness refers to the *structure and quality* of social integration—the feeling of belonging—perceived social support is the *subjective appraisal* of the specific emotional, informational, and instrumental resources available within that network (Cohen and Wills, 1985; Lakey and Cohen, 2000). For university students, navigating a period of significant transition, establishing strong social connections (social connectedness) is a critical resource for fostering resilience, self-esteem, and emotional well-being (Lee and Robbins, 1998).

The benefits of social connectedness in higher education are well-documented. Tinto (1975) seminal model highlights social integration as a core predictor of a student’s commitment and persistence. Modern research confirms this, showing that students with strong connections to peers and faculty demonstrate greater academic success and are less likely to drop out (Allen et al., 2008; Freeman et al., 2007). This finding is particularly critical in the Chinese context, where studies have linked poor peer relations and unsupportive classroom environments to both “hidden” and actual student dropout (Gao et al., 2019; Gan et al., 2023). Strong social relationships also buffer against academic stressors and bolster mental health, with low connectedness being a known risk factor for depression and anxiety (Cacioppo et al., 2015; Poole et al., 2023). These findings establish social connectedness as a crucial asset, but they also raise the critical question of *how* these connections translate into the psychological and behavioral resources needed for effective coping.

While the university environment offers fertile ground for forming new relationships, it also presents significant challenges to deep connection, such as intense academic competition and the difficulty of integrating into new social landscapes (Fisher et al., 2013; Hausmann et al., 2007). A student’s success in navigating this environment determines the quality of their social network, which in turn forms the structural basis for social support.

In our model, social connectedness functions as the direct antecedent to perceived social support. The two constructs must be distinguished. Social connectedness refers to the structure and quality of a student’s social integration—the feeling of belonging. In contrast, perceived social support is the subjective appraisal of the specific emotional, informational, and instrumental resources available *within* that network (Cohen and Wills, 1985; Lakey and Cohen, 2000). A student who feels highly connected has more opportunities to form trusting relationships with peers, faculty, and mentors. These relationships, in turn, foster the belief that tangible help and emotional encouragement are available if needed. In this way, social connectedness creates the relational infrastructure from which a student’s perception of support is derived.

### 2.2 Optimism: conceptual foundations and influences

Optimism, defined as a generalized expectation of positive outcomes (Scheier and Carver, 1985; Seligman, 1991), is a significant internal resource that strongly influences student well-being and resilience. Rooted in both dispositional traits and learned attributions (Carver et al., 2010; Seligman et al., 1995), a positive future outlook is consistently linked to persistence, motivation, and goal-oriented behavior in the face of academic pressures (Chemers et al., 2001; Rand et al., 2011).

The benefits of optimism for students are broad. Academically, optimistic students tend to achieve higher grades and demonstrate greater perseverance (Richardson et al., 2012). Psychologically, optimism is a robust protective factor against depression, anxiety, and burnout (Rasmussen et al., 2009; Scheier et al., 1994). Within the scope of our model, **however,** the crucial question is *how* this internal disposition translates into effective coping behaviors. The literature suggests that optimism does not function in a vacuum; rather, it actively shapes how students engage with their environment and its challenges.

Fredrickson (2001) broaden-and-build theory provides the theoretical foundation for optimism’s role in our model. This theory posits that positive emotions and expectancies broaden an individual’s cognitive and behavioral repertoires, encouraging them to build lasting personal resources. Optimism thus fosters a proactive coping style (Scheier and Carver, 1985), where students are more likely to actively confront problems. This proactive orientation extends to the social domain: optimistic individuals tend to initiate social connections, expect positive outcomes from social interactions, and view seeking help as an effective problem-solving strategy, not a sign of weakness. Research by Brissette et al. (2002) directly supports this pathway, demonstrating that optimism plays a significant role in social network development and the mobilization of social support during life transitions. An optimistic student is therefore more likely to perceive their social environment as a source of willing and available support.

While the benefits of optimism are broadly supported, its manifestation can be shaped by contextual factors. Research suggests, for instance, that the expression and impact of optimism may differ across cultural contexts (Chang et al., 2003). Specifically, within the Chinese context, optimism may be less about an individualistic, dispositional positive outlook and more intertwined with a pragmatic belief in the value of persistence and effort, often in service of collective or familial goals (Cheng and Hamid, 1997). This “realistic optimism” may be a particularly powerful motivator in the face of the intense academic competition characteristic of Chinese universities. Systemic inequities faced by first-generation or minority students can also present significant challenges to maintaining a positive outlook (Terenzini et al., 1996). Acknowledging these factors is important, yet the underlying psychological process remains robust: a hopeful orientation encourages engagement with, rather than withdrawal from, available resources.

### 2.3 Adaptive coping: strategies and contextual influences

Adaptive coping comprises the cognitive, emotional, and behavioral strategies used to effectively manage stressors and promote well-being (Carver and Scheier, 1994; Lazarus and Folkman, 1984). In our study, it serves as the key functional outcome. This repertoire includes problem-focused strategies like proactive planning, emotion-focused strategies like cognitive reappraisal, and, most central to our model, the active seeking of social support (Compas et al., 2001; Lazarus and Folkman, 1984; Skinner et al., 2003).

The importance of adaptive coping is evident in its strong links to both academic success and mental well-being. Problem-focused strategies like goal-setting are associated with higher academic engagement and performance (Vizoso et al., 2018; Zimmerman, 2002), while traits like “grit” predict retention (Duckworth et al., 2007). On the mental health front, active coping strategies are linked to reduced psychological distress (Flett et al., 2016; Meng and D'Arcy, 2016), and cognitive reappraisal can lessen the burden of academic stress (Gross, 2002). Conversely, maladaptive styles such as avoidance and rumination are associated with burnout and depression (Nolen-Hoeksema et al., 2008; Tanner et al., 2015). Indeed, internal processes like negative repetitive thinking and maladaptive emotional beliefs have been shown to be key mechanisms that link negative interpersonal experiences, such as emotional abuse, to psychological distress (Rezaei et al., 2025b).

The university years are a particularly critical period for developing these skills, as students face new and complex stressors (Baker and Siryk, 1999). During this transition, mobilizing social resources becomes a paramount coping strategy. Proactive support-seeking is a hallmark of adaptive coping in this population and is associated with smoother academic and social adjustment (Pritchard et al., 2007). This specific strategy—turning to one’s social network for help—represents the behavioral activation of perceived social support, directly linking the mediator to the outcome in our model. However, the *expression* of these strategies is influenced by contextual factors (Evans and Kim, 2013). Cultural background is particularly salient; for example, in collectivist-influenced settings like China, individuals may be more likely to employ strategies like emotional suppression to maintain group harmony (Wei et al., 2013). Furthermore, while proactive support-seeking is key, in this context, it may be enacted more indirectly to avoid burdening others and to preserve “face” (Chang, 2015). This cultural nuance underscores the potential importance of *perceived* support—the belief that help is available even if not explicitly sought—as a critical precursor to coping.

### 2.4 Perceived social support: dimensions and effects

As defined previously, perceived social support is the subjective evaluation of available aid (Cobb, 1976; Cohen and Wills, 1985), and it serves as the central mediating mechanism in our model. Crucially, it is this *perception* of support, rather than its objective availability, that most significantly impacts well-being (Lakey and Cohen, 2000). We therefore conceptualize this subjective appraisal as the active ingredient linking antecedents to outcomes. It functions as a logical bridge, representing the psychological product of a student’s social connectedness (Tinto, 1975) while also being shaped by their dispositional optimism, as previously discussed.

The theoretical basis for this link is the well-established stress-buffering hypothesis (Cohen and Wills, 1985), which posits that the *belief* in a supportive network mitigates the negative effects of stress (Turner and Brown, 2010). For students, this perception provides the emotional, informational, and instrumental resources to manage academic and personal challenges (Cutrona et al., 1994). A meta-analysis by Chu et al. (2010) confirmed this strong link between perceived support and well-being in young people. Accordingly, students who feel supported by peers and faculty are more engaged, perform better academically, and are less likely to drop out (Derakhshan and Fathi, 2024; Hausmann et al., 2007; Pittman and Richmond, 2008). This sense of support directly enables adaptive coping; for instance, a supported student is more likely to seek help rather than turn to maladaptive behaviors like avoidance or problematic smartphone use (Yang et al., 2019).

Although the stress-buffering effect is robust, the sources and effectiveness of this support are shaped by contextual factors. Students draw support from diverse sources, including family, peers, and institutional agents (Reeve et al., 2013). The alignment of this support with a student’s specific needs and cultural background is crucial for its success (Calvete and Connor-Smith, 2006; Frías et al., 2014). Indeed, research highlights that in collectivist-influenced cultures, there may be a tendency to underutilize explicit support-seeking to avoid relational burden or “face loss” (Chang, 2015), and these cultural values can moderate the association between internal states and coping behaviors (Frías et al., 2014). Moreover, systemic barriers can create disparities in perceived support for marginalized students (Brondolo et al., 2009; Woodford et al., 2018). Furthermore, the *quality* of social feedback is critical; it is not just the presence of support but the absence of *invalidation* that matters. Recent work highlights that perceived emotional invalidation can act as a potent moderator, exacerbating the link between negative life experiences (like emotional abuse) and emotion dysregulation, which in turn predicts psychological distress (Rezaei et al., 2025a). The relevance and psychometric soundness of measuring perceived emotional invalidation have also been established in non-Western cultural contexts (Rezaei et al., 2024). These factors form the complex backdrop against which the mediation process unfolds.

### 2.5 Conceptual framework and hypotheses

Based on the preceding review, we propose an integrated conceptual framework to guide this study. Our framework synthesizes two key psychological theories—the stress-buffering model of social support (Cohen and Wills, 1985) and Fredrickson’s (2001) broaden-and-build theory—to explain the process through which foundational resources are converted into adaptive coping. We posit that social connectedness (an external, relational asset) and optimism (an internal, dispositional asset) act as key antecedents that foster a student’s perceived social support. This perception of support, in turn, functions as the direct psychological mechanism that enables the use of adaptive coping strategies. The stress-buffering model explains the direct link between perceived social support and adaptive coping, while the broaden-and-build theory helps explain how optimism facilitates the development of perceived social support.

**Figure 1:**
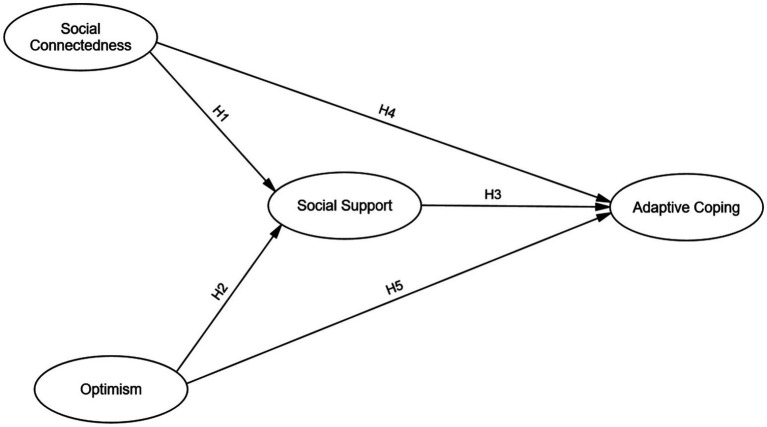
The hypothesized mediation model.

The hypothesized relationships among these four variables are visualized in our conceptual framework (Figure 1).

Based on this integrated framework, we formulated the following specific hypotheses to be tested in the quantitative phase of the study:

Hypothesis 1 (*H1*): Social connectedness will be positively associated with perceived social support.

Hypothesis 2 (*H2*): Optimism will be positively associated with perceived social support.

Hypothesis 3 (*H3*): Perceived social support will be positively associated with adaptive coping.

Hypothesis 4 (*H4*): Social connectedness will be positively associated with adaptive coping (direct effect).

Hypothesis 5 (*H5*): Optimism will be positively associated with adaptive coping (direct effect).

Hypothesis 6 (*H6*): Perceived social support will mediate the positive relationship between social connectedness and adaptive coping.

Hypothesis 7 (*H7*): Perceived social support will mediate the positive relationship between optimism and adaptive coping.

This study employs a mixed-methods design to test these hypotheses. The quantitative phase will rigorously test the paths specified in the model using structural equation modeling. The subsequent qualitative phase will then explore the lived experiences behind these statistical relationships, providing a deeper, more contextualized understanding of the psychosocial dynamics that support student well-being and adaptive success.

## 3 Methods and materials

This study employed an explanatory sequential mixed-methods design (Creswell and Plano Clark, 2017) to investigate the mediating role of perceived social support in the relationship between social connectedness and optimism on adaptive coping among university students in mainland China. The quantitative phase, conducted first, examined the hypothesized mediation model using survey data. The qualitative phase, which followed, aimed to provide a deeper understanding of the quantitative findings through semi-structured interviews.

### 3.1 Participants

A total of 463 undergraduate students (232 females, 231 males) from three major universities in mainland China participated in the study. Participants were recruited through convenience sampling from psychology, sociology, and education-related courses. The age of participants ranged from 18 to 25 years (*M* = 20.32, *SD* = 1.87). The majority of participants were Han Chinese (95.25%), which is representative of the general university student population in mainland China, with the remaining 4.75% identifying with various ethnic minority groups such as Uyghur, Tibetan, and Mongolian. Participants were primarily full-time undergraduate students enrolled in diverse academic disciplines, including humanities (35.42%), social sciences (40.17%), and natural sciences (24.41%), reflecting a broad spectrum of the university student body. The majority of participants were in their second and third year of study (65.01%), with the remainder being first and fourth-year students (34.99%). Approximately 70.20% of the participants resided in on-campus dormitories, while the remaining 29.80% lived off-campus, either in rented apartments or with family. To ensure diversity in perspectives, efforts were made to recruit students from both urban (62.42%) and rural (37.58%) backgrounds, reflecting the diverse geographical distribution of students across mainland China. Prior to participation, informed consent was obtained from all students, and participation was voluntary with no direct incentives offered, although students were informed that their participation would contribute to a better understanding of student well-being and coping strategies.

We conducted an *a priori* power analysis using a Monte Carlo simulation approach, recommended for detecting indirect effects (Fritz and MacKinnon, 2007; Muthén and Muthén, 2002). Using Mplus (Muthén and Muthén, 1998-2017), we specified our hypothesized mediation model. Based on prior research, we aimed to detect small-to-medium indirect effects, setting standardized path coefficients for the predictor → mediator (*α*) path at approximately 0.25 and the mediator → outcome (*β*) path at approximately 0.20. We ran 10,000 replications to simulate the data. Results indicated that a sample size of approximately *N* = 350 would achieve statistical power of 0.80 (at *α* = 0.05) for detecting the smallest hypothesized indirect effect using bias-corrected bootstrap confidence intervals. Thus, our final sample of *N* = 463 provides sufficient power for testing the proposed mediational pathways.

### 3.2 Procedure

The university’s Research Ethics Committee approved the study (Approval No. BUAS-SS-2024-037) before data collection. Quantitative data for this study were collected from October to November 2024. Following the initial statistical analysis of the survey data, the qualitative phase was conducted in February 2025 to ensure the interview questions were directly informed by the quantitative findings.

Initially, participants completed a paper-based questionnaire during class. Trained research assistants distributed the questionnaires, provided a brief introduction, and assisted with the subsequent data entry under the author’s supervision. The questionnaire, which took 30–40 min to complete, included measures of social connectedness, optimism, perceived social support, and adaptive coping. Research assistants were available for procedural questions but did not clarify questionnaire content to avoid influencing responses. Completed forms were collected immediately.

Following quantitative data analysis, 20 participants were purposively selected for semi-structured interviews. To achieve maximum variation in our qualitative sample, we first categorized the full sample of participants into tertiles (low, medium, high) based on their scores for each of the four quantitative variables. We then purposively recruited 20 individuals who represented a wide range of score profiles, including those with consistently high or low scores across variables, as well as those with mixed profiles (e.g., high optimism but low social connectedness). A summary of the quantitative scores for these 20 interview participants is provided in Table 1, demonstrating the achieved variation across all measures. The goal was to ensure a diverse range of experiences and viewpoints within the 20-person sample, not to cover every possible combination of scores.

**Table 1:** Descriptive statistics for interview participant subsample (*N* = 20).

Participant ID	Social connectedness	Optimism	Perceived social support	Adaptive coping
P1	5.80	3.83	6.75	3.71
P2	2.15	1.17	2.50	1.93
P3	4.30	3.00	5.42	2.86
P4	5.50	1.67	6.17	2.21
P5	2.45	3.67	3.25	3.50
P6	4.85	3.50	6.58	3.64
P7	3.90	2.83	5.17	3.00
P8	3.30	2.17	4.08	2.50
P9	5.10	3.33	6.25	3.29
P10	2.90	2.00	3.83	2.29
P11	5.95	3.17	6.92	3.43
P12	2.00	1.50	2.17	2.07
P13	4.50	2.50	5.75	2.79
P14	5.65	3.00	6.50	3.14
P15	3.50	1.83	4.50	2.64
P16	4.15	3.17	4.83	3.07
P17	5.20	3.83	6.42	3.57
P18	2.60	2.33	3.00	2.43
P19	4.70	2.67	5.92	2.93
P20	3.45	1.83	3.75	2.57
Subsample mean	4.20	2.65	4.99	2.88
Subsample SD	1.26	0.78	1.56	0.54
Full sample mean	4.21	2.95	5.23	2.88
Full sample SD	0.78	0.63	1.12	0.45

All scores are reported as item averages to allow for comparison across scales. The full sample statistics (*N* = 463) from Table 2 are included for reference. The subsample demonstrates wide variation across the full range of scores for each variable.

The interview protocol was developed from the quantitative findings to explore the “how” and “why” behind the observed relationships. The guide used open-ended questions and probes to elicit detailed responses about social connectedness, optimism, social support, and coping mechanisms in the context of university life. Example questions included: “Could you describe what social connectedness means to you?” and “How does your general outlook on the future influence how you deal with challenges?” Crucially, the protocol also included specific probes designed to elicit narratives about how these psychosocial factors influenced one another in students’ experiences, allowing us to explore the processes underlying the statistical model. A complete list of the guiding questions used in the semi-structured interviews is provided in Appendix A.

Prior to the interviews, the author underwent a two-day training workshop on qualitative interviewing techniques and ethics. Interviews, lasting 45–60 min, were conducted in Mandarin Chinese in private on-campus rooms. All interviews were audio-recorded and transcribed verbatim. Initial transcription was performed using automated transcription software and then manually reviewed and corrected by the author to ensure complete accuracy. A bilingual researcher translated the transcripts into English, and a second bilingual researcher verified the accuracy of the translations via back-translation of a subset of quotes. Minor adjustments were made as necessary to maintain fidelity and nuance.

### 3.3 Instruments

This study employed self-report questionnaires to evaluate participants’ social connectedness, optimism, perceived social support, and adaptive coping mechanisms. All instruments utilized Likert-type scales and have demonstrated robust psychometric properties in prior research. As the original scales were developed in English, we undertook a rigorous, committee-based translation and back-translation procedure to ensure the linguistic, cultural, and conceptual equivalence of all measures for our Mandarin-speaking Chinese sample.

The adaptation process for each scale involved the following five steps: (1) Two independent bilingual experts, both native Mandarin speakers, translated the original English items into Simplified Chinese. (2) The research team, along with the two translators, convened to compare these initial translations, discuss any discrepancies, and produce a single, reconciled Chinese version. (3) A third bilingual expert, who was not familiar with the original English scales, independently translated the reconciled Chinese version back into English. (4) The research team then compared the back-translated English version with the original scale to check for semantic equivalence and resolve any inconsistencies. (5) Finally, the preliminary Chinese versions of all questionnaires were pilot-tested with a group of 30 undergraduate students, who were not part of the main study. During this pilot phase, students were asked about the clarity and comprehensibility of each item, and minor wording adjustments were made based on their feedback to improve naturalness and ensure all constructs were understood as intended.

To confirm the construct validity of the adapted scales within our specific sample, we conducted a series of Confirmatory Factor Analyses (CFAs) using AMOS 26.0. We tested the established theoretical factor structures for each of the primary scales. The 12-item MSPSS demonstrated excellent fit for its original three-factor structure (Family, Friends, Significant Other): *χ^2^*(48) = 95.21, *p* < 0.001, *CFI* = 0.98, *TLI* = 0.97, and *RMSEA* = 0.045 [90% CI 0.031, 0.059]. The 6-item LOT-R also showed strong fit for its single-factor structure (with the 4 filler items excluded): *χ^2^*(9) = 16.45, *p* = 0.06, *CFI* = 0.99, *TLI* = 0.98, and *RMSEA* = 0.042 [90% CI 0.000, 0.078]. The 20-item SCS-R showed an acceptable fit for its original two-factor structure (Connectedness and Disconnectedness): *χ^2^*(169) = 388.70, *p* < 0.001, *CFI* = 0.95, *TLI* = 0.94, and *RMSEA* = 0.053 [90% CI 0.046, 0.060]. These CFA results provide strong evidence for the measurement validity of the adapted scales in this study. In addition to construct validity, internal consistency was assessed using Cronbach’s alpha. All scales showed good to excellent reliability: SCS-R (*α* = 0.88), LOT-R (*α* = 0.82), MSPSS (*α* = 0.91), and the Brief COPE adaptive subscales composite (*α* = 0.85).

#### 3.3.1 Social connectedness scale-revised (SCS-R)

Social connectedness was evaluated using the Social Connectedness Scale-Revised (SCS-R) (Lee and Robbins, 1995). Following the adaptation procedure described above, the final 20-item measure was used to gauge an individual’s subjective sense of interpersonal closeness and belonging. The scale incorporates both positively and negatively framed statements to capture diverse aspects of social connectedness, including intimacy and feelings of connection versus isolation. Respondents indicated their level of agreement with each statement using a 6-point Likert scale, ranging from 1 (Strongly disagree) to 6 (Strongly agree). Higher scores reflect greater social connectedness.

#### 3.3.2 Revised life orientation test (LOT-R)

Optimism was assessed with the Revised Life Orientation Test (LOT-R) (Scheier et al., 1994). The adapted Chinese version of this 6-item scale (accompanied by 4 filler items) was used to measure dispositional optimism, conceptualized as generalized positive expectations for the future. Participants rated their agreement with each statement on a 5-point Likert scale ranging from 0 (Strongly disagree) to 4 (Strongly agree). Higher scores indicate a greater degree of optimism.

#### 3.3.3 Multidimensional scale of perceived social support (MSPSS)

Perceived social support was measured via the Multidimensional Scale of Perceived Social Support (MSPSS) (Zimet et al., 1988). The adapted Chinese version of this 12-item scale assesses an individual’s perception of social support availability from three distinct sources: Family, Friends, and Significant Other. Participants rated each item using a 7-point Likert scale, ranging from 1 (Very strongly disagree) to 7 (Very strongly agree). Higher scores indicate greater perceived social support.

#### 3.3.4 Brief COPE inventory—adaptive coping subscales

Adaptive coping strategies were evaluated using specific subscales from the Brief COPE Inventory (Carver, 1997). This study focused on the seven adaptive coping subscales. The adapted Chinese versions of these items were administered to the participants. Participants rated each item on a 4-point scale ranging from 1 (“I have not been doing this at all”) to 4 (“I’ve been doing this a lot”). A composite adaptive coping score was calculated, with higher scores indicating greater utilization of adaptive coping strategies.

### 3.4 Data analysis

Descriptive statistics and Pearson correlations were calculated for all study variables. To test the hypothesized mediation model, path analysis was conducted using Structural Equation Modeling (SEM) in AMOS 26.0 (Arbuckle, 2019). Social connectedness and optimism were specified as predictors, perceived social support as the mediator, and adaptive coping as the outcome variable. Model fit was evaluated using the chi-square statistic (*χ^2^*), Comparative Fit Index (*CFI*), Tucker-Lewis Index (*TLI*), Root Mean Square Error of Approximation (*RMSEA*), and Standardized Root Mean Square Residual (*SRMR*). Acceptable fit was defined by conventional thresholds: *CFI* and *TLI* values >0.90, *RMSEA* ≤ 0.08, and *SRMR* ≤ 0.08 (Hu and Bentler, 1999). A bootstrapping procedure with 5,000 resamples was employed to test the significance of indirect effects.

Before analysis, we screened the data for missing values and outliers. Little’s MCAR test confirmed that missing data (<5%) were completely at random [*χ*^2^(14) = 18.76, *p* = 0.178], allowing for imputation using the expectation–maximization (EM) algorithm. The assumptions of normality, linearity, and homoscedasticity were also assessed and met.

For the qualitative data, we used thematic analysis following Braun and Clarke's (2006) six-phase approach. This involved familiarizing ourselves with the data, generating initial codes, searching for and reviewing themes, and then defining and naming them. Using an inductive approach, we developed codes from recurring patterns in the data related to participants’ experiences. To ensure rigor, the author and a second independent coder (a doctoral student with training in qualitative methods) independently coded a subset of three transcripts (15% of the data). The coders then met to compare codes, discuss discrepancies, and refine the coding framework until a satisfactory level of inter-coder reliability was achieved (Cohen’s Kappa > 0.80). The author then applied the finalized coding framework to the remaining transcripts. The qualitative findings were integrated with the quantitative results in the discussion to provide a comprehensive, nuanced understanding of the relationships between the study variables.

## 4 Findings

### 4.1 Quantitative results

Preliminary analyses were conducted to ensure the data met the assumptions for SEM. Little’s MCAR test showed missing data were completely at random [*χ*^2^(14) = 18.76, *p* = 0.178]. With missing values under 5% and the MCAR assumption met, expectation–maximization (EM) imputation was used. Inspection of scatterplots and residual plots, along with non-significant Shapiro–Wilk tests for normality (all *p*s > 0.05) and a non-significant Breusch-Pagan test for homoscedasticity (*p* = 0.23), confirmed the assumptions of normality, linearity, and homoscedasticity. Outliers identified via Mahalanobis distance showed no cases above the critical value of *p* < 0.001. For *N* = 463, these tests are sensitive, but skewness and kurtosis values in Table 2 (all within ±1.0) further supported the assumption of normality.

**Table 2:** Descriptive statistics for study variables (*N* = 463).

Variable	*M*	*SD*	Range	Skewness	Kurtosis
Social connectedness (SCS-R)	4.21	0.78	1.80–6.00	−0.32	0.15
Optimism (LOT-R)	2.95	0.63	0.00–4.00	−0.18	−0.25
Perceived social support (MSPSS)	5.23	1.12	1.00–7.00	−0.55	0.62
Adaptive coping (Brief COPE)	2.88	0.45	1.57–4.00	−0.05	−0.10

Table 2 summarizes descriptives for social connectedness, optimism, perceived social support, and adaptive coping; scores are item averages to facilitate comparison across scales. The mean age was 20.32 years (*SD* = 1.87), typical for undergraduates. The Social Connectedness Scale-Revised (SCS-R) mean was 4.21 (*SD* = 0.78), showing moderate connectedness. The Revised Life Orientation Test (LOT-R) mean for optimism was 2.95 (*SD* = 0.63), above the scale midpoint, indicating a generally optimistic outlook. The Multidimensional Scale of Perceived Social Support (MSPSS) mean was 5.23 (*SD* = 1.12), suggesting strong perceived support from family, friends, and others. The Brief COPE adaptive subscales mean was 2.88 (*SD* = 0.45), indicating moderate use of adaptive strategies.

Pearson correlations checked links among variables (Table 3). All showed significant positive ties (*p* < 0.001), as expected. Social connectedness had moderate correlation with optimism (*r* = 0.42, 95% CI [0.34, 0.50], *p* < 0.001), strong with perceived social support (*r* = 0.51, 95% CI [0.44, 0.58], *p* < 0.001), and weak-to-moderate with adaptive coping (*r* = 0.35, 95% CI [0.27, 0.43], *p* < 0.001). Optimism linked moderately to perceived social support (*r* = 0.38, 95% CI [0.30, 0.46], *p* < 0.001) and weakly to adaptive coping (*r* = 0.28, 95% CI [0.20, 0.36], *p* < 0.001). Perceived social support and adaptive coping correlated moderately (*r* = 0.45, 95% CI [0.38, 0.52], *p* < 0.001). These sizes indicate moderate-to-strong ties, with higher scores in one often matching others, yet some independent variation.

**Table 3:** Bivariate correlations among study variables (*N* = 463).

Variable	1	2	3	4
1. Social Connectedness (SCS-R)	--			
2. Optimism (LOT-R)	0.42**	--		
3. Perceived Social Support (MSPSS)	0.51**	0.38**	--	
4. Adaptive Coping (Brief COPE)	0.35**	0.28**	0.45**	--

**p < 0.001. Confidence Intervals (CI) are reported at 95%.

To rigorously test the hypothesized mediation model, full structural equation modeling (SEM) was conducted using AMOS 26.0 (Arbuckle, 2019). Each construct was modeled as a latent variable indicated by its items or subscales (e.g., SCS-R: 20 items; LOT-R: 6 scored items; MSPSS: 12 items; Brief COPE adaptive subscales: 14 items). This method addressed measurement error and estimated relations among latent constructs. The model specified covariances between exogenous latent variables (social connectedness and optimism) and all disturbance terms, without cross-loadings or correlated errors. Social connectedness and optimism served as exogenous predictors, perceived social support as the mediator, and adaptive coping as the endogenous outcome.

The model showed good fit. The *χ*^2^ statistic was significant [*χ*^2^(15) = 35.21, *p* = 0.002], but this test is sample-size sensitive and often significant with large N, so other indices guided evaluation (Kline, 2016). The Comparative Fit Index (CFI = 0.97) and Tucker-Lewis Index (TLI = 0.96) surpassed 0.95, signaling excellent fit. The Root Mean Square Error of Approximation (RMSEA = 0.06, 90% CI [0.04, 0.08]) and the Standardized Root Mean Square Residual (SRMR = 0.045) fell well below the 0.08 threshold, reinforcing good fit. Together, these indices confirm the model adequately represents the data’s covariance structure.

**Figure 2:**
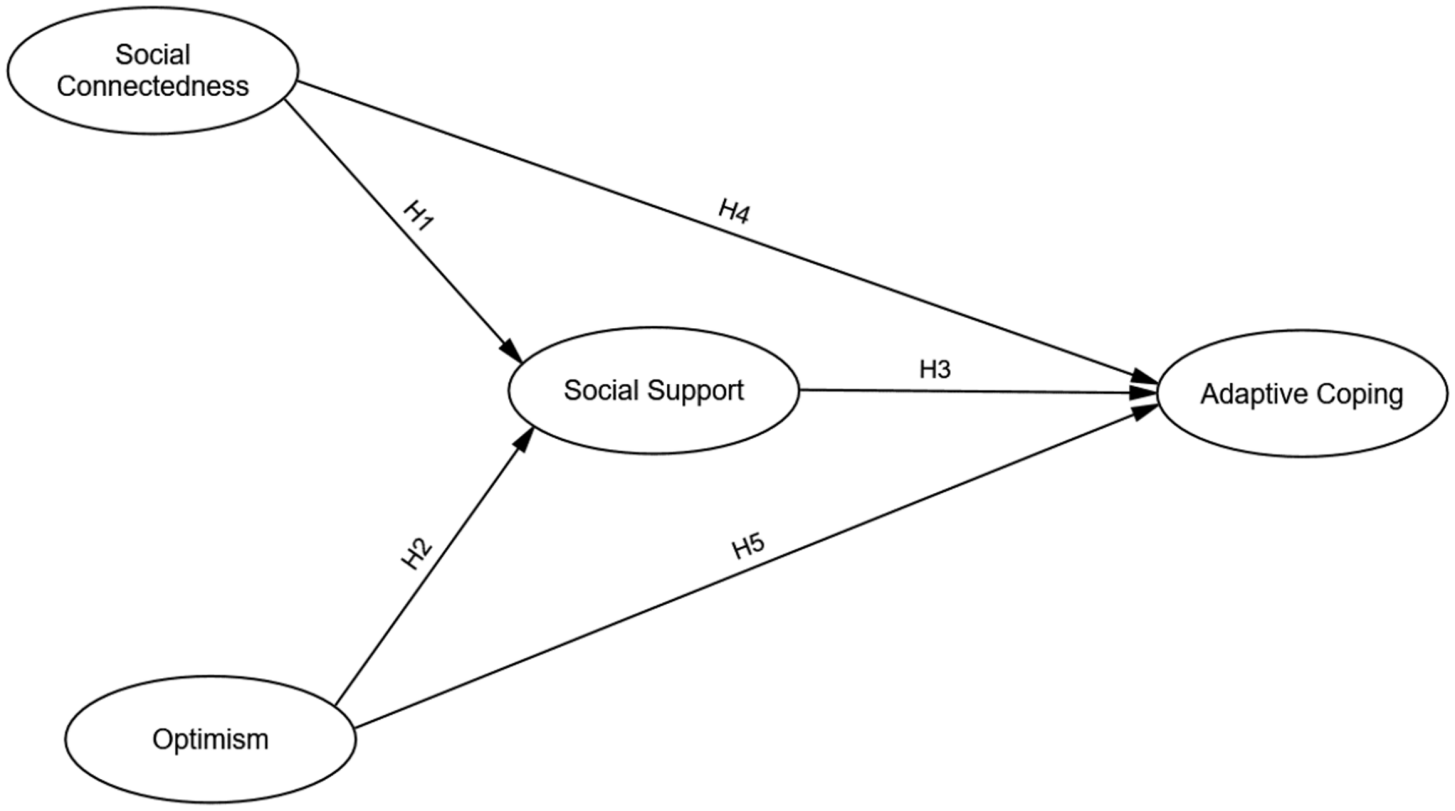
The mediation model of social connectedness and optimism predicting adaptive coping through perceived social support. Standardized path coefficients are presented. *p < 0.05, **p < 0.01, ***p < 0.001.

Figure 2 displays standardized path coefficients (*β*). Social connectedness had significant positive direct effects on perceived social support (*β* = 0.38, *SE* = 0.05, *p* < 0.001) and adaptive coping (*β* = 0.18, *SE* = 0.07, *p* = 0.009). Optimism positively predicted perceived social support (*β* = 0.25, *SE* = 0.06, *p* < 0.001) and adaptive coping (*β* = 0.12, *SE* = 0.05, *p* = 0.042). Perceived social support positively predicted adaptive coping (*β* = 0.30, *SE* = 0.06, *p* < 0.001). These coefficients indicate stronger direct effects for social connectedness and perceived social support, while optimism’s effects are weaker yet significant.

To test mediation effects, bootstrapping with 5,000 resamples estimated indirect effects and confidence intervals. Social connectedness had a significant indirect effect on adaptive coping via perceived social support (*β* = 0.11, 95% CI [0.07, 0.15], *p* < 0.001). Optimism showed a similar significant indirect effect (*β* = 0.08, 95% CI [0.04, 0.12], *p* < 0.001). These effects, with CIs excluding zero, support perceived social support’s mediating role between both social connectedness and optimism with adaptive coping. Direct effects of social connectedness (*β* = 0.18, *p* < 0.01) and optimism (*β* = 0.12, *p* < 0.05) on adaptive coping stayed significant after including indirect paths, indicating partial mediation. This means perceived social support acts as a key route for these influences, but direct links persist, suggesting that other unmeasured mechanisms are also at play. For instance, the remaining direct path from optimism, though small in magnitude, may reflect its more direct cognitive influence on coping strategies (e.g., positive reframing) that operate separately from social processes.

The model explained variance in endogenous variables as well. It accounted for 32% in perceived social support (*R*^2^ = 0.32) and 28% in adaptive coping (*R*^2^ = 0.28). These *R*-squared values reflect moderate model strength in predicting both outcomes (Table 4).

**Table 4:** Path coefficients for the hypothesized mediation model.

Path	*β*	*SE*	*p*	95% CI	Effect type
Social connectedness → Perceived social support	0.38	0.05	<0.001	[0.28, 0.48]	Direct
Social connectedness → Adaptive coping	0.18	0.07	0.009	[0.04, 0.32]	Direct
Optimism → Perceived social support	0.25	0.06	<0.001	[0.13, 0.37]	Direct
Optimism → Adaptive coping	0.12	0.05	0.042	[0.02, 0.22]	Direct
Perceived social support → Adaptive coping	0.30	0.06	<0.001	[0.18, 0.42]	Direct
Indirect effects					
Social connectedness → Perceived social support → Adaptive coping	0.11	–	<0.001	[0.07, 0.15]	Indirect
Optimism → Perceived social support → Adaptive coping	0.08	–	<0.001	[0.04, 0.12]	Indirect

β = Standardized path coefficient, SE = Standard Error, CI = Confidence Interval. Indirect effects were estimated using bootstrapping with 5,000 resamples.

### 4.2 Qualitative results

The qualitative phase aimed to provide a rich, contextualized understanding of the quantitative mediation model. Thematic analysis of 20 semi-structured interviews yielded findings that interrogate the *processes* suggested by the quantitative model. The analysis moves beyond simply describing students’ experiences with each variable to illustrate how these factors appear to interact. Table 1 provides the quantitative scores for the 20 students who participated in the interviews, demonstrating the variation achieved through our purposive sampling strategy.

To move beyond simple description and directly investigate the model’s mechanisms, we presented the analysis to align with the quantitative pathways. The first three themes illustrate the mediational process, from the antecedents to the mediator, and from the mediator to the outcome, drawing primarily on cases that align with the model. The final theme critically examines cases that appear to contradict the dominant model, providing a more nuanced interpretation and potential explanations for the partial mediation observed quantitatively.

#### 4.2.1 Theme 1: social connectedness as the foundation for perceived support

This theme directly illustrates the process underlying the Social Connectedness → Perceived Social Support pathway (*β* = 0.38 in the quantitative model). Participants explained that the initial act of forming bonds—in dorms, clubs, or classes—was the necessary first step that built the “relational infrastructure” for support. These established relationships provided a sense of trust and safety that transformed a general network into a perceived source of aid.

Participant 3 (a female humanities student) described this foundation: “University was a fresh start… especially in my dorm. We were all in the same boat… so it was easy to connect… We would stay up late just talking about our anxieties and excitement.”

This foundation of shared experience was what students later drew upon, as Participant 14 (a male social sciences student; High SC = 5.65, High PSS = 6.50) explicitly linked: “My friends here are like my university family… Because we are so close, I never feel weird asking for help. I know they’ll be there. Knowing I have friends who understand exactly what I’m going through makes a huge difference.” In this way, social connectedness (the “close” feeling) appears to create the psychological resource of perceived support (“I know they’ll be there”).

#### 4.2.2 Theme 2: optimism as a catalyst for mobilizing social support

This theme provides a process-oriented view of the Optimism → Perceived Social Support pathway (*β* = 0.25 in the quantitative model). Participants’ narratives suggested that optimism did not just co-exist with support but actively unlocked it. An optimistic outlook was described as a proactive force that lowered the psychological barrier to seeking help, reframing it as an effective, positive strategy rather than a sign of weakness.

Participant 1 (a male psychology student; High OPT = 3.83, High PSS = 6.75) described this proactive stance: “If I encounter a problem. I immediately start thinking about solutions. Being optimistic helps me to be proactive and not just give up or feel sorry for myself.”

This proactive approach clearly extended to the social domain. Participant 6 (a female education student; High OPT = 3.50, High PSS = 6.58) provided a perfect illustration of this mediational link: “Because I’m optimistic, I’m not afraid to ask for help when I need it. I believe that people will be willing to assist me, and that seeking help is a sign of strength, not weakness. This positive expectation makes it easier to reach out.” This quote powerfully illustrates the potential process: the disposition (optimism) leads directly to a positive appraisal of social resources (perceiving support as willing and available).

#### 4.2.3 Theme 3: activating perceived support into adaptive coping

This theme completes the mediation model, illustrating the Perceived Social Support → Adaptive Coping pathway (*β* = 0.30 in the quantitative model). Participants confirmed that the perception of support was the functional resource that enabled them to engage in specific coping behaviors. The most frequently cited adaptive strategy was the act of seeking support—a direct behavioral activation of the psychological resource.

Participant 11 (a female social sciences student; High PSS = 6.92, High AC = 3.43) stated this firmly: “When I’m really stressed… talking to my friends or family always helps. Just sharing my worries… and knowing they are there for me, listening without judgment, makes me feel so much better and more able to cope. It’s like a weight lifted off my shoulders.”

Similarly, Participant 4 (a male humanities student; High PSS = 6.17, Low AC = 2.21, discussed further in Theme 4) noted how this extends beyond peers: “Seeking advice from professors or older students who have been through similar experiences is also a crucial coping strategy for me. They offer valuable insights and guidance.” In both cases, the *belief* that support was available and effective (Perceived Social Support) was described as a direct trigger for engaging in the *behavior* of seeking help (Adaptive Coping), although other factors could interfere, as discussed next.

#### 4.2.4 Theme 4: complicating the model—contradictory cases and alternative pathways

A purely confirmatory analysis risks oversimplifying reality. Our purposive sampling included students whose experiences did not neatly fit the primary mediation model, providing critical insight into alternative pathways and the model’s limitations, as suggested by the partial mediation in the quantitative results.

##### 4.2.4.1 Subtheme 4.1: the direct path—high optimism bypassing social support

We critically examined Participant 5, whose profile contradicts the main mediation model: he scored high on optimism (3.67) and adaptive coping (3.50) but relatively low on social connectedness (2.45) and perceived social support (3.25). This case appears to directly illustrate the quantitative model’s significant direct path from optimism to coping (β = 0.12). Participant 5 described himself as highly self-reliant:

“I’m a positive person, yes. I believe I can solve my own problems. When I get a bad grade, I do not really talk to people about it; I just see it as a chance to learn. I make a detailed plan and I follow it. I trust myself to handle it. Relying on others can just get complicated.”

For Participant 5, optimism did not manifest primarily as a social tool but rather as high self-efficacy or an internal locus of control. His coping was highly intrapersonal (e.g., planning, positive reframing), suggesting his resilience operates largely independently of the social mediation pathway our main model proposed. This provides a compelling narrative explanation for the remaining direct effect observed in the SEM.

##### 4.2.4.2 Subtheme 4.2: a broken link—when high support does not lead to coping

Conversely, Participant 4 presented a puzzle: high social connectedness (5.50) and high perceived support (6.17) did not translate into high adaptive coping (2.21). His low optimism (1.67) provided a potential explanation, challenging the PSS → Coping link. He felt supported, but his pessimistic outlook seemed to prevent him from activating that support effectively:

“Oh, my friends are great. They always ask me how I’m doing and offer to help me study. [High PSS]. But honestly, I just feel like I’m bothering them. I’ll say ‘I’m fine’ even when I’m not. I just end up worrying about the exam on my own, thinking I’ll probably fail anyway, so what’s the point of their help? [Low Optimism]. I just end up watching videos or sleeping [Low Coping].”

This narrative suggests that high perceived support may not be sufficient for adaptive coping if dispositional factors like pessimism intervene. It points toward a potential *moderating* effect, where low optimism negates the functional benefits of high perceived social support by inhibiting the behavioral activation (help-seeking) needed for coping. This case highlights the complexity missed by a simple mediation model.

Overall, the qualitative results provide a vivid, process-oriented illustration that both confirms and complicates the quantitative model. While the experiences of participants like P6 and P14 bring the mediational pathways to life, the analysis of contradictory cases (e.g., Participants 4 and 5) is crucial. It provides grounded potential explanations for the partial mediation found in the SEM results, highlighting the importance of direct pathways (e.g., optimism-as-self-efficacy leading to intrapersonal coping) and potential moderators (e.g., pessimism inhibiting the activation of support) not captured in the simple mediation model. In this way, the qualitative findings contextualize the statistical patterns and provide a deeper, more critical understanding of how these psychosocial factors function together.

### 4.3 Integration of quantitative and qualitative findings

In line with our explanatory sequential design, this section integrates the quantitative results from the mediation model with the qualitative themes derived from the interviews. The goal is to use the rich, contextual narratives from students to illustrate potential mechanisms behind the statistical relationships observed in the SEM model and explore complexities suggested by the data. This integration provides a deeper meta-inference about the psychosocial processes associated with adaptive coping.

The central quantitative finding was that perceived social support significantly mediated the relationships between both social connectedness and optimism with adaptive coping. The qualitative data offer a lens through which to view these statistical associations, illustrating plausible lived experiences behind the pathways, while also highlighting important nuances. A joint display table (Table 5) summarizes this integration, followed by a narrative explanation.

**Table 5:** Joint display of quantitative and qualitative findings.

Quantitative finding (What?)	Illustrative qualitative data (How?)	Meta-inference (Why it matters)
Pathway 1: Social connectedness → Perceived social support → Adaptive coping (*β* = 0.11, *p* < 0.001)	Theme: From social connectedness to activated support. Students described how established friendships created a trusted foundation for seeking help. Quote (Participant 14): “My friends here are like my university family… Because we are so close, I never feel weird asking for help…”	The mediation suggests it’s not just about having a network, but about having a *trusted* network. Social connectedness appears to provide the relational safety and opportunity that transforms the abstract potential for support into the concrete, proactive coping behavior of seeking help.
Pathway 2: Optimism → Perceived social support → Adaptive coping (*β* = 0.08, *p* < 0.001)	Theme: From optimism to seeking support. Students explained that a positive outlook lowered the psychological barrier to asking for help, framing it as a strength. Quote (Participant 6): “Because I’m optimistic, I’m not afraid to ask for help… seeking help is a sign of strength…”	The mediation suggests an agentic process. Optimism may act as a psychological catalyst empowering students to actively mobilize social resources. This potentially reframes help-seeking from a passive last resort into a proactive strategy, offering one explanation for how an internal disposition is associated with coping through social means.

#### 4.3.1 Explaining the mediation pathways and partial mediation

The first significant indirect effect showed that social connectedness fosters adaptive coping through perceived social support. The qualitative data illustrate this potential process by showing that connection can be a necessary precondition for support mobilization. As Participant 14 explained, the feeling of having a “university family” (high social connectedness) removes the psychological friction (“I never feel weird”) associated with asking for help. This suggests that the statistical path may rely on the trust and shared understanding built within established relationships. Students seem to leverage their sense of belonging to activate their support network, which then becomes a primary tool for coping.

The second significant indirect effect revealed that optimism also facilitates adaptive coping through perceived social support. The qualitative findings provide a powerful illustration for this less intuitive pathway. Participant 6’s narrative vividly depicts optimism functioning as a proactive mindset. Her belief that others would be willing to help and her framing of help-seeking as a “sign of strength” directly mirror the statistical link. This meta-inference suggests that mediation is not necessarily a passive process where support simply happens to optimists. Instead, optimism appears to encourage the behavior of seeking support, empowering students to transform their social environment into a tangible resource. This provides a clear, process-oriented perspective on how an internal, dispositional asset might be converted into an external, social coping strategy.

Critically, the quantitative model (Figure 2) found partial mediation, not full mediation. Significant direct paths remained from both social connectedness (*β* = 0.18) and optimism (*β* = 0.12) to adaptive coping. This indicates that perceived social support is not the only pathway linking these variables. Our qualitative analysis, particularly through the purposive sampling and examination of contradictory cases (Theme 4), helps to contextualize and offer potential explanations for this remaining variance.

First, the direct path from optimism to coping was vividly illustrated by Participant 5. This student’s profile (High Optimism, High Coping; Low SC, Low PSS) and narrative (“I believe I can solve my own problems… I make a detailed plan… I trust myself”) strongly suggest an intrapersonal pathway where optimism facilitates coping directly through mechanisms like self-efficacy, planning, and positive reframing, operating independently of perceived social support.

Second, the analysis of cases like Participant 4 (High SC, High PSS; Low Optimism, Low Coping) challenges the universality of the PSS → Coping link and suggests a more complex interaction. His narrative (“I know my friends are there… But… I’ll probably fail anyway, so what’s the point…”) implies that optimism might also function as a moderator. High perceived support may only translate into adaptive coping when a student possesses a sufficient level of optimism to activate those resources. This potential moderation effect offers another explanation for why PSS does not fully account for the relationship between the antecedents and coping.

In sum, the integration of our quantitative and qualitative findings provides a cohesive and critical picture. The statistical model identifies the significant pathways. The narratives from confirming cases (like P14 and P6) illustrate how these pathways might plausibly function, showing that connectedness can provide the trusted opportunity for support and optimism can provide the psychological impetus to seize it. Furthermore, the analysis of contradictory cases (like P5 and P4) provides critical insights into the partial mediation, suggesting that optimism can also facilitate intrapersonal coping and, in its absence, may inhibit the activation of social support.

## 5 Discussion

The central finding of this mixed-methods study is that perceived social support serves as a crucial psychological mechanism that is statistically associated with how the benefits of social connectedness and optimism relate to adaptive coping among university students. Our integrated findings suggest that it is not merely the presence of social ties or a positive outlook that fosters resilience, but the subjective perception of a supportive network that is a key correlate of that resilience. This discussion unpacks the theoretical and practical implications of this mediation model, using our qualitative meta-inferences to contextualize these statistical associations and add nuance to existing models of student resilience.

Our descriptive and correlational findings provide the necessary context for this main conclusion. The data depicted a student population with a moderate sense of social connectedness and a generally optimistic outlook, yet a high level of perceived social support. This landscape, vividly described in our qualitative themes, reflects the complex reality of university life—a “crucible” of both social opportunity and isolating academic pressure (Tanner et al., 2015). It is within this dynamic environment that the process of converting foundational resources into effective coping unfolds. The significant correlations between all variables confirmed that these constructs are meaningfully related, setting the stage for testing the specific pathways of influence.

The core contribution of this study lies in unpacking the indirect effects that define the mediation model. The significant pathway from social connectedness through perceived social support to adaptive coping (*β* = 0.11) adds a critical antecedent condition to the classic stress-buffering model (Cohen and Wills, 1985). While that model masterfully explains how perceived support protects against stress, our integrated findings offer a process-oriented perspective on how that perception might be built. It is not a passive state but is founded on the relational infrastructure of social connectedness. This moves beyond simply stating that “friends are helpful” and specifies a plausible process: social integration appears to build the perceived safety net that students draw upon to manage stress. As Participant 14 articulated, having a “university family” (social connectedness) gave him the confidence to seek help because the close bond made it feel safe and natural. While our cross-sectional data cannot prove causality, this qualitative evidence provides a compelling, grounded illustration of the statistical path. This suggests that theoretical models of stress and coping should not treat perceived support as a given starting point but as a psychological resource that must first be cultivated through genuine connection.

Perhaps the most novel finding is the mediational role of perceived social support in the relationship between optimism and adaptive coping (*β* = 0.08). This finding provides a crucial, process-oriented extension to Fredrickson (2001) broaden-and-build theory. While the theory posits that positive states build personal resources, our qualitative data illustrate a plausible behavioral mechanism through which this occurs in the social domain: optimism appears to foster an *agentic mindset* that reframes help-seeking as a proactive strength. As Participant 6 so clearly explained, “Because I’m optimistic, I’m not afraid to ask for help,” viewing it as a sign of strength. This moves beyond the general link found by Brissette et al. (2002) to specify that optimism does not just passively correlate with larger networks; it may also actively encourage students to mobilize those networks as coping resources. The theoretical implication is that optimism’s effect on coping is not just cognitive (e.g., positive reframing), but also behavioral, by prompting the social actions that unlock support.

By serving as a common mediator for both an external resource (social connectedness) and an internal one (optimism), perceived social support emerges as a central psychological nexus in our model. Our findings suggest it is the critical point of convergence where both structural-relational assets and internal-dispositional assets both statistically connect with the tangible practice of adaptive coping. This challenges a view of these resources as operating in parallel and instead proposes a more integrated, convergent model where the subjective feeling of being supported is a key gateway to effective action. This positions perceived social support not just as another variable, but as a primary target for intervention.

Finally, the finding of partial mediation warrants discussion. The significant direct paths from both social connectedness and optimism to adaptive coping suggest that perceived social support is a major pathway, but not the only one. Our qualitative data, particularly the analysis of contradictory cases, offer compelling alternative explanations for these remaining effects. The direct path from optimism to coping (*β* = 0.12), for instance, was illustrated by Participant 5, a student with high optimism and high coping but low social connectedness and support. His narrative suggested that his optimism manifested as a high degree of self-efficacy: “I believe I can solve my own problems… I make a detailed plan and I follow it. I trust myself to handle it.” This suggests the direct path represents a more intrapersonal, self-reliant form of coping (e.g., positive reframing, planning), which operates independently of the social mediation pathway (Chemers et al., 2001). Conversely, the case of Participant 4, who had high perceived support but low optimism and low coping, suggests a potential moderation effect. His pessimism (“I’ll probably fail anyway, so what’s the point…”) prevented him from activating the support he knew he had. This implies that optimism may not only function as a predictor, but also as a necessary moderator for the Support → Coping pathway to function effectively. These alternative explanations highlight important avenues for future research using more complex moderated-mediation models.

In summary, the findings of this study converge to form a cohesive picture of how psychosocial resources work together to promote student resilience. By elevating the mediation analysis and integrating it with rich qualitative data, we have shown that social connectedness and optimism are associated with adaptive coping in large part by statistically linking to students’ perception of available social support. This process-oriented model not only validates existing frameworks but enriches them by providing a more dynamic, integrated understanding of how students may leverage their internal and external resources into resilience.

## 6 Conclusion and implications

This mixed-methods study provides compelling evidence of the intricate psychosocial dynamics that are associated with adaptive coping in university students. Our findings demonstrate that perceived social support is a critical mediating mechanism statistically linking both social connectedness and optimism with students’ ability to manage stress. The quantitative model, contextualized by our qualitative narratives, reveals that fostering connectedness and optimism is not just beneficial on its own, but also because these resources are associated with students’ perceptions of their support networks. This highlights the synergistic power of these three constructs in fostering resilience during the formative university years. Our research moves beyond a deficit-focused approach to student well-being by emphasizing the proactive cultivation of psychosocial strengths.

This research extends the stress-buffering model of social support (Cohen and Wills, 1985) by highlighting its potential as a mediator between broader psychosocial constructs like social connectedness and optimism, and coping outcomes. This suggests that interventions should target upstream factors that enhance perceived social support, not just direct coping skills. The findings also support Fredrickson’s broaden-and-build theory (2001) by illustrating how optimism’s influence extends into the social sphere, plausibly shaping students’ perception of available support and thereby associating with adaptive coping. Future research should use longitudinal designs to explore the dynamic interplay of these constructs over time.

Beyond theory, our findings have significant practical implications for higher education institutions. However, generic solutions are insufficient. Our mediation results suggest a more targeted approach is necessary. First, to enhance well-being, universities should implement initiatives that do not just create opportunities for connection (social connectedness) but that are explicitly designed to strengthen the *perception* of support. For instance, structured peer mentoring programs (Oddone Paolucci et al., 2021) should train mentors to move beyond passive friendship and actively communicate their availability and non-judgmental stance, thereby solidifying the mentee’s perceived support network. Second, our findings on optimism suggest interventions should focus on reframing the *act* of help-seeking. As our qualitative data (e.g., Participant 6) illustrated, optimistic students were more likely to view seeking help as a “sign of strength.” Therefore, wellness programs, such as cognitive behavioral workshops (Seligman, 1991), should not just aim to build general positive thinking, but should explicitly frame help-seeking as a proactive, high-competence strategy for success, rather than a remedial action. Finally, these interventions must be culturally attuned. In the Chinese context, where concerns about “face” and group harmony can create a reluctance to burden others (Chang et al., 2003), promoting direct, explicit help-seeking may be ineffective. A more innovative approach would be to create low-barrier, low-risk platforms for perceiving support, such as structured group-based problem-solving workshops (where no single student is ‘the problem’) or anonymous online Q&A platforms with trained peer leaders and faculty. These strategies would allow students to feel supported (PSS) without incurring the potential social cost of direct help-seeking, thus bypassing a key cultural barrier.

## 7 Limitations and suggestions for future research

This study offers valuable insights, but its limitations must be considered when interpreting the findings and guiding future inquiry. Two major methodological constraints must be strongly emphasized. The primary and most significant limitation is the cross-sectional design. While our mediation model is grounded in theory, the data are correlational and preclude any causal inferences. We cannot confirm the hypothesized *direction* of the pathways. It is plausible, for instance, that students who are adept at coping subsequently perceive more social support and are more successful at building social connections. Longitudinal research is not just a suggestion; it is essential to validate the mediational *process* we have proposed, particularly during key transitions like university entry and graduation.

This constraint is compounded by the exclusive reliance on self-report measures. While we used validated scales and our CFA results provided support for their structure, these data are susceptible to bias. Variables such as optimism and adaptive coping are particularly prone to social desirability bias. Furthermore, collecting all measures from the same source at the same time introduces the significant risk of common method variance, which may have artificially inflated the strength of the observed relationships between constructs. Future studies must use multi-method assessment strategies, such as behavioral observations, peer ratings, or physiological measures of stress, to provide a more objective evaluation.

Other limitations also warrant consideration. The study’s findings are limited by the specific sample, which was drawn from three universities in one region of China. This may limit the broad generalizability of our results to other cultural contexts and educational systems where norms, academic pressures, and coping styles can differ. Replicating this study in diverse cultural and institutional settings would be valuable. Additionally, our exclusive focus on the adaptive coping subscales from the Brief COPE inventory, though targeted, did not capture the full spectrum of coping responses. Future research should include both adaptive and maladaptive coping strategies to develop a more complete understanding of student coping repertoires. Finally, while we identified perceived social support as a significant mediator, our finding of partial mediation (and the analysis of contradictory qualitative cases) suggests other unmeasured variables are at play. We encourage future research to explore other potential mediators and moderators. For instance, cognitive-emotional factors, such as meta-cognitive beliefs or negative repetitive thinking, may function as additional serial mediators in the link to psychological distress (Rezaei et al., 2025b). Similarly, future models should test the potential moderating role of negative interpersonal feedback, such as perceived emotional invalidation (Rezaei et al., 2025a), in addition to self-efficacy, emotion regulation, and the potential moderating role of optimism on the support-coping link, as suggested by our qualitative analysis. A more complete model that accounts for these variables would provide a more nuanced understanding of the psychosocial factors that promote student well-being.

## Data Availability

The raw data supporting the conclusions of this article will be made available by the authors, without undue reservation.

## Appendix A: semi-structured interview protocol

The following questions served as a guide for the semi-structured interviews. The interviewer used follow-up probes (e.g., "Can you tell me more about that?", "How did that make you feel?", "What happened next?") to encourage participants to elaborate on their experiences.

### Part 1: introduction and general university experience

1. To start, could you please tell me a bit about your overall experience as a university student so far? What have been some of the highlights and some of the challenges?

### Part 2: social connectedness

2. What does the term "social connectedness" or "feeling a sense of belonging" mean to you in the context of university life?
3. Can you describe your main friendships or social groups here at the university? How did these connections form?
4. Are there times when you feel particularly connected to others on campus? Conversely, are there situations that make you feel disconnected or lonely?

### Part 3: optimism and mindset

5. Generally speaking, when you think about your future, what comes to mind? Would you describe yourself as more of an optimist or a pessimist?
6. Think about a recent academic or personal challenge you faced. How did you think about the situation at the time?
7. How does your general outlook on life influence how you handle day-to-day stressors, like a heavy workload or a difficult assignment?

### Part 4: social support

8. When you are feeling stressed or overwhelmed, who are the first people you turn to for support? (Probes: Family? Friends here at university? Friends from home? Professors?)
9. What kind of support is most helpful for you? Is it practical advice, emotional encouragement, or just having someone listen? Can you give an example?
10. Can you describe a time when you felt really well-supported by someone during a difficult period at university?

### Part 5: adaptive coping

11. Think about a recent stressful period, like exam season. What specific things did you do to manage that stress and get through it?
12. How do you try to balance your academic responsibilities with your social life and personal well-being? What strategies do you use?

### Part 6: Integration and process questions (exploring mediation)

13. How do your friendships and social connections here [Social Connectedness] affect the way you deal with academic pressure [Adaptive Coping]?
14. Does your general outlook [Optimism] influence whether or not you reach out to others for help when you need it [Social Support]? How so?
15. Can you walk me through a specific time when talking to a friend or family member [Social Support] helped you to solve a problem or feel better about a stressful situation [Adaptive Coping]?

### Part 7: closing

1. 16. Is there anything else you would like to share about your experiences with stress, relationships, or coping at university that we haven't talked about today?
